# NLRP3 inflammasome and Sjögren disease: mechanisms, clinical associations and therapeutic prospects

**DOI:** 10.3389/fimmu.2026.2004934

**Published:** 2026-10-30

**Authors:** Rui Niu, Qiwei Li, Linwei Li, Qingai Guo, Jiayi Zhao, Xiadong Yang, Shouze Ren, Lina Zhao, Chang Liu, Ying Ma, Peng Zhou, Jingyuan Li, Zeguang Li, Hua Liang

**Affiliations:** 1 Heilongjiang University of Chinese Medicine, Harbin, China; 2 Heilongjiang Provincial Hospital, Harbin, China; 3 The Second Hospital of Heilongjiang Province, Harbin, China; 4 The First Affiliated Hospital of Heilongjiang University of Chinese Medicine, Harbin, China

**Keywords:** glandular inflammation, NLRP3 inflammasome, pyroptosis, Sjögren disease, therapeutic strategies

## Abstract

Sjögren disease (SjD; formerly referred to as Sjögren syndrome) is a chronic autoimmune disease characterized by exocrine gland dysfunction and systemic immune abnormalities. In recent years, clinical and preclinical studies have suggested that dysregulation of the NOD-like receptor family pyrin domain-containing 3 (NLRP3) inflammasome may contribute to inflammatory responses, glandular damage, and disease progression in SjD, although the available human evidence remains primarily associative. This review summarizes alterations in the NLRP3 inflammasome in patients with SjD, animal models, and cell models; discusses its upstream regulation, downstream effects, associated clinical phenotypes, and systemic involvement; and compares evidence across different cell types, disease stages, and therapeutic interventions. Based on current evidence, NLRP3 is more likely to function as an inflammatory amplification and regulatory node in the context of pre-existing autoimmunity and tissue stress, with its role varying according to cell type, disease status, and the local microenvironment. NLRP3 and its related pathways therefore have potential therapeutic value; however, evidence for direct targeting of NLRP3 in SjD remains largely limited to preclinical studies, whereas some immunomodulatory drugs supported by clinical studies are not NLRP3-specific therapies. Further clarification of the cell-specific and stage-specific roles of NLRP3, together with identification of patient subgroups that may benefit, will be critical for promoting its clinical translation.

## 1 Introduction

Sjögren disease (SjD; formerly known as Sjögren syndrome) is a chronic systemic autoimmune disease characterized by chronic lymphoepithelial inflammation and dysfunction of exocrine glands, particularly the salivary and lacrimal glands (1). Women are affected substantially more often than men, with a female-to-male ratio of approximately 9:1 to 20:1, and the peak age at onset is 40–50 years (2, 3). The prevalence of SjD varies considerably depending on the study population, classification criteria, and study design. Because previous epidemiological studies were mainly based on the traditional classification framework of primary Sjögren syndrome (pSS), unified global prevalence data reassessed entirely according to the new SjD nomenclature remain unavailable. The 2023 International Rome Consensus, published in 2025, reported that the prevalence of Sjögren disease in previous studies ranged from approximately 1 to 72 per 10,000 population (0.01%–0.72%), with substantial variation among studies (1). In epidemiological studies focusing on pSS, the prevalence in the general population is generally estimated to be approximately 0.01%–0.05% (2). The clinical manifestations of SjD are highly heterogeneous. In addition to exocrine gland manifestations such as dry mouth and dry eyes, SjD can involve multiple organs and systems, including the musculoskeletal system, kidneys, lungs, and peripheral nervous system (3). SjD can significantly impair patients’ quality of life and is associated with an increased risk of B-cell non-Hodgkin lymphoma (B-NHL), which has important implications for long-term prognosis (3, 4).

The development and progression of SjD involve the combined effects of genetic susceptibility, epigenetic alterations, environmental factors, and immune dysregulation. Genome-wide association studies have identified several genetic loci associated with SjD susceptibility, including the HLA region and genes such as *STAT4* and *IRF5*. In addition, epigenetic alterations, including DNA methylation and histone modifications, are also considered to contribute to disease development and progression (5). At the immunopathological level, aberrant activation of B and T cells, autoantibody production, and sustained release of multiple pro-inflammatory cytokines collectively contribute to glandular inflammation and tissue damage (6, 7). Notably, salivary gland epithelial cells are not merely targets of immune attack but also actively participate in local immune and inflammatory responses by secreting cytokines and chemokines, recruiting and regulating immune cells, and sustaining the inflammatory microenvironment (8). Although understanding of the pathogenesis of SjD has advanced substantially in recent years, current clinical management still mainly focuses on alleviating sicca symptoms and controlling systemic involvement. In patients with systemic organ involvement, glucocorticoids and other immunomodulatory or immunosuppressive therapies may be used; however, treatment options capable of durably restoring exocrine gland function or effectively modifying the disease course remain limited (3, 9). Therefore, further elucidating the molecular mechanisms underlying persistent immune-mediated inflammation and glandular damage in SjD is important for identifying novel therapeutic targets and advancing the development of disease-modifying therapeutic strategies.

Inflammasomes are multiprotein complexes composed of intracellular pattern-recognition receptors, adaptor proteins, and effector proteins, and serve as important molecular platforms linking innate immune sensing to inflammatory responses (10). Among them, the NOD-like receptor family pyrin domain-containing 3 (NLRP3) inflammasome is one of the most extensively studied inflammasomes. Its activation promotes caspase-1 activation and the maturation of IL-1β and IL-18, and can induce gasdermin D-mediated pyroptosis, thereby amplifying local inflammatory responses and tissue damage (11, 12). In recent years, alterations in the NLRP3 inflammasome and its related effector molecules have been observed in the salivary glands, peripheral immune cells, and ocular surface tissues of patients with SjD. Further mechanistic evidence has also been obtained from animal and cell models, suggesting that dysregulation of the NLRP3 inflammasome may contribute to inflammatory amplification, glandular damage, and disease progression in SjD. However, current human studies still provide primarily associative evidence, whereas more direct causal and interventional evidence is derived mainly from preclinical models. Therefore, the precise pathological role of the NLRP3 inflammasome in SjD and its clinical value as a therapeutic target remain to be further established.

Although the potential relationship between the NLRP3 inflammasome and Sjögren disease (SjD) has been discussed in previous reviews, evidence from patient samples, animal models, and cell models has continued to accumulate in recent years. At the same time, substantial heterogeneity among studies in terms of tissue source, disease stage, experimental model, and intervention outcome has become increasingly apparent. In particular, evidence of NLRP3-related alterations in human SjD remains largely associative, while the effects of NLRP3-targeted interventions are not fully consistent across different preclinical models, suggesting that the role of NLRP3 may depend on disease stage and cellular context. Therefore, this review summarizes the available evidence from patients with SjD, animal models, and cell models, with a focus on alterations in NLRP3 expression, mechanisms of inflammasome activation, pathological significance, and NLRP3-targeted therapeutic strategies, aiming to provide a more objective assessment of the role of the NLRP3 inflammasome in SjD and its potential for clinical translation.

## 2 Literature search strategy

This review employed a narrative literature search strategy using PubMed, Web of Science, Scopus, and the China National Knowledge Infrastructure (CNKI), with a focus on Chinese- and English-language literature published between September 2016 and September 2026. The last search was conducted in September 2026, and representative earlier studies were additionally identified through reference tracking. The English search terms included “Sjögren disease,” “Sjögren syndrome,” “primary Sjögren syndrome,” “NLRP3,” “inflammasome,” and “pyroptosis.” The Chinese search terms included “干燥病,” “干燥综合征,” “NLRP3炎症小体,” and “细胞焦亡.

Regarding study selection, priority was given to human studies, animal model studies, and cell-based experiments directly related to NLRP3 inflammasome abnormalities, upstream regulation, downstream effects, and therapeutic interventions in SjD. Representative mechanistic studies and high-quality reviews were also included to provide general biological background. Studies involving only other diseases that did not provide direct or necessary background support for NLRP3-related mechanisms in SjD, as well as studies lacking key information, duplicate reports, or studies with limited relevance to the topic of this review, were not included as primary evidence. Throughout the review, SjD-specific evidence was distinguished from mechanistic evidence derived from other diseases or from general NLRP3 biology to avoid presenting mechanistic extrapolation as direct evidence in SjD.

## 3 The NLRP3 inflammasome

NLRP3 is an important member of the NOD-like receptor (NLR) family and functions primarily as a cytoplasmic pattern-recognition receptor that senses a variety of pathogen-associated molecular patterns and damage-associated molecular patterns. The NLRP3 protein consists of an N-terminal pyrin domain (PYD), a central NACHT domain, and C-terminal leucine-rich repeats (LRRs). The canonical NLRP3 inflammasome is mainly composed of NLRP3, apoptosis-associated speck-like protein containing a CARD (ASC), and the effector protein pro-caspase-1. The ATPase activity of the NACHT domain and NLRP3 oligomerization are important for inflammasome assembly (11, 13).

Activation of the canonical NLRP3 inflammasome generally involves two stages: priming and activation. During the priming stage, Toll-like receptors and other pattern-recognition receptors activate NF-κB signaling, thereby promoting the expression of NLRP3 and pro-IL-1β. Subsequently, stimuli such as extracellular ATP, K^+^ efflux, mitochondrial dysfunction, and lysosomal damage can further trigger NLRP3 activation, and the interaction between NIMA-related kinase 7 (NEK7) and NLRP3 is an important step in inflammasome assembly (14, 15). Activated NLRP3 recruits ASC and pro-caspase-1, promotes caspase-1 activation, and further processes pro-IL-1β and pro-IL-18 to generate mature IL-1β and IL-18 (11).

In addition to transcriptional regulation, the NLRP3 inflammasome is also regulated by various post-translational modifications, including phosphorylation, ubiquitination, and SUMOylation, as well as by organelle homeostasis. These mechanisms can affect NLRP3 protein stability, subcellular localization, ATPase activity, and oligomerization, thereby collectively determining the extent of inflammasome activation (16). Therefore, the NLRP3 inflammasome is not a simple “on-off” system but is subject to multilayered dynamic regulation.

Following activation of the NLRP3 inflammasome, caspase-1 promotes the maturation of IL-1β and IL-18 and cleaves gasdermin D (GSDMD), releasing its pore-forming N-terminal fragment. GSDMD-N forms pores in the plasma membrane, facilitating inflammatory mediator release and pyroptosis (17, 18). NLRP3 is expressed in myeloid cells, including monocytes, macrophages, dendritic cells, and neutrophils, and has also been reported in certain lymphoid and non-immune cells. Sustained or excessive inflammasome activation can promote chronic inflammation, inflammatory cell death, and tissue damage, providing a biological basis for investigating its pathological role in SjD (19).

## 4 NLRP3 Inflammasome and SjD

Inflammation and immune dysregulation are important pathological features underlying the development and progression of SjD. In recent years, abnormalities in the NLRP3 inflammasome and related molecules have been reported in salivary glands, peripheral immune cells, ocular surface tissues, and various preclinical models, suggesting that the NLRP3 inflammasome may contribute to local inflammatory amplification, glandular tissue damage, and disease progression (20–22). However, findings across studies are not fully consistent with respect to tissue source, measured indicators, and changes in NLRP3 itself. Therefore, it cannot yet be concluded that widespread and consistent systemic NLRP3 hyperactivation occurs in SjD. This section examines alterations in the NLRP3 inflammasome in SjD and their potential pathological significance from both clinical and preclinical perspectives.

### 4.1 Clinical evidence

Existing clinical studies have identified NLRP3 inflammasome-related abnormalities in the major target glands, ocular surface, and peripheral immune system of patients with SjD (**Table 1**). Baldini et al. found increased *NLRP3* and *CASP1* mRNA expression in labial salivary glands from patients with SjD compared with non-SjD sicca controls, whereas *PYCARD* (ASC) mRNA showed no significant difference; IL-18 protein levels in saliva were also increased (20). Kim et al. further observed increased expression of ASC, caspase-1, and IL-1β, as well as elevated levels of activated caspase-1 and mature IL-1β, in peripheral blood mononuclear cells (PBMCs) from patients with SjD; however, *NLRP3* mRNA itself showed no significant change (21). These findings suggest that abnormalities in inflammasome-related components and downstream effector molecules do not necessarily indicate consistent upregulation of NLRP3 itself. Meanwhile, Niu et al. observed increased expression of NLRP3 and caspase-1 in the conjunctival epithelium, accompanied by elevated tear levels of IL-1β and IL-18, in patients with SjD-related dry eye diagnosed according to the 2002 American-European Consensus Group classification criteria (23). It should be noted that this study used the 2002 classification criteria that were current at the time, rather than the subsequently established 2016 American College of Rheumatology/European League Against Rheumatism (ACR/EULAR) classification criteria.

**Table 1:** NLRP3 inflammasome components and related molecules in patients with Sjögren disease.

Study population	Sample size	Comparator	Sample / tissue	Main molecular findings	Clinical phenotype / associations	Key limitations	Reference
Primary SjD	Labial glands: 21 SjD / 15 non-SjD sicca; 6 additional preserved parotid specimens used as controls in selected assays	Non-SjD sicca for core molecular comparisons; normal controls for selected additional assays	Labial SGs; saliva; normal parotid tissue in selected assays	NLRP3: mRNA in labial SGs ↑; ASC: mRNA in labial SGs, NS; caspase-1: mRNA in labial SGs ↑; pro-caspase-1 protein in labial SGs ↑; IL-18: protein in saliva ↑	Glandular NLRP3 mRNA positively correlated with FS (r = 0.532; P = 0.041)	Small sample size; some normal tissue controls were parotid specimens, whereas SjD specimens were labial glands	20
SjD-associated dry eye	54 SjD-associated dry eye / 50 non-SjD dry eye / 46 healthy controls	Healthy controls for the main comparisons; additional non-SjD dry eye group	CIC samples; tears	NLRP3: mRNA in conjunctival cells ↑; protein by WB ↑; caspase-1: mRNA in conjunctival cells ↑; pro-caspase-1 and caspase-1 proteins in conjunctival cells ↑; IL-1β: conjunctival mRNA ↑; tear protein concentration ↑; IL-18: conjunctival mRNA ↑; tear protein concentration ↑	Reduced tear film stability; reduced tear secretion; increased ocular surface staining	ASC not examined; WB samples pooled from 6 subjects	23
Primary SjD	33 SjD / 34 healthy controls; all women	Age- and sex-matched healthy controls	PBMCs; serum	NLRP3: mRNA in PBMCs, NS; ASC: mRNA in PBMCs ↑; caspase-1: mRNA in PBMCs ↑; IL-1β: mRNA in PBMCs and serum protein concentration ↑; IL-18: mRNA in PBMCs, NS; serum protein concentration ↑	Generally low disease activity (median ESSDAI: 2); IL-1β mRNA associated with disease damage; serum IL-18 correlated with SSDDI	Cross-sectional study; potential medication effects	21
Primary SjD stratified by germinal center-like structures and subsequent MALT lymphoma	Glandular analysis: 45 SjD / 25 non-SjD sicca; 5 patients subsequently developed MALT lymphoma	Non-SjD sicca; additional comparisons between SjD subgroups	Labial SGs; saliva	NLRP3: mRNA in labial SGs ↑; caspase-1: mRNA in labial SGs ↑; IL-1β: mRNA in labial SGs ↑; IL-18: mRNA in labial SGs ↑	Higher baseline expression in patients who subsequently developed MALT lymphoma; expression associated with glandular inflammation, anti-SSA positivity and selected lymphoma risk features	ASC not examined; only 5 lymphoma events; inconsistent sample counts in the original report	24
Primary SjD with low or high lymphoma risk and MALT lymphoma subgroups	PBMCs qPCR: 39 SjD / 16 healthy controls; serum sample counts varied by analyte; glands: 14 SjD / 9 non-SjD controls	PBMCs and serum: healthy controls; glands: non-SjD controls	PBMCs; serum; SGs	NLRP3: mRNA in PBMCs ↑; ASC: mRNA in PBMCs and serum protein concentration ↑; proportion of ASC-positive macrophages in SGs ↑; ASC specks observed; caspase-1: mRNA in PBMCs ↑; IL-1β: serum protein concentration ↑; IL-18: serum protein concentration ↑	Serum ASC and IL-18 positively correlated with cumulative ESSDAI; abnormalities more pronounced in high-risk and lymphoma subgroups	Different participant subsets used for different assays	25
Primary SjD	Glandular qPCR: 18–20 SjD / 7–8 sicca controls; serum / saliva ELISA: 80 SjD / 20 healthy controls; saliva WB: 50 SjD / 14 sicca controls / 14 healthy controls	Glands: non-SjD sicca; serum and saliva ELISA: healthy controls; saliva WB also included sicca controls	MSGs; serum; saliva	NLRP3: glandular mRNA, NS; ASC: glandular mRNA, NS; caspase-1: glandular mRNA ↑; protein in glandular epithelial cells ↑; salivary protein concentration and cleaved caspase-1 ↑; serum protein concentration, NS; IL-1β: glandular mRNA and serum and salivary protein concentrations, NS; IL-18: glandular mRNA ↑; serum and salivary protein concentrations ↑	Glandular caspase-1 expression positively correlated with expression of type I IFN-related genes	Direct human comparisons of NLRP3 and ASC mainly based on mRNA; comparator types differed across samples	26
Primary SjD	10 SjD / 10 healthy controls, as reported by the authors	Healthy individuals undergoing routine health examinations	Samples derived from fasting peripheral blood	NLRP3: protein concentration ↑; ASC: protein concentration ↑; caspase-1: protein concentration ↑; IL-1β: protein concentration ↑	2016 ACR/EULAR criteria; untreated or off medication for >3 months	Reported sex counts inconsistent with the sample size; blood fraction not specified in the methods	27 (USP5)
Primary SjD stratified by FS	Total cohort: 25 SjD / 11 sicca controls, all women; WB: 9 SjD (FS ≤2: 4; FS >2: 5) / 5 sicca controls	Non-SjD sicca	Whole-tissue lysates of labial SGs	NLRP3: protein ↑ in the FS >2 subgroup; NS in the FS ≤2 subgroup; caspase-1: cleaved protein ↑ in both FS subgroups; IL-18: protein ↑ in both FS subgroups	Glandular IL-18 protein positively correlated with FS; NLRP3 differences depended on FS subgroup	No human comparisons of ASC or IL-1β; small WB sample size	28

↑, increased relative to the stated comparator; NS, no statistically significant difference; mRNA expression and protein levels or concentrations are recorded separately.

ACR, American College of Rheumatology; ASC, apoptosis-associated speck-like protein containing a CARD; CARD, caspase recruitment domain; CIC, conjunctival impression cytology; ELISA, enzyme-linked immunosorbent assay; ESSDAI, EULAR Sjögren’s Syndrome Disease Activity Index; EULAR, European Alliance of Associations for Rheumatology; FS, focus score; IFN, interferon; IL, interleukin; MALT, mucosa-associated lymphoid tissue; MSGs, minor salivary glands; PBMCs, peripheral blood mononuclear cells; qPCR, quantitative polymerase chain reaction; SGs, salivary glands; SjD, Sjögren disease; SSA, Sjögren’s syndrome-related antigen A; SSDDI, Sjögren’s Syndrome Disease Damage Index; WB, western blotting.

In addition to abnormalities in core inflammasome components, alterations in pyroptosis and downstream effector molecules have also been observed in glandular tissues from patients with SjD. Hong et al. observed increased expression of caspase-1, IL-18, and GSDMD in minor salivary gland epithelial cells from patients with SjD (26). Zhang et al. also reported abnormal expression of GSDMD and other pyroptosis-related molecules in salivary gland tissues from patients with SjD (29). In addition, Parisis et al. reported enhanced IL-18-related signaling in the serum and salivary gland tissues of patients with SjD (30). These findings support the presence of inflammasome-related effects and pyroptotic abnormalities in SjD glands. However, caspase-1, IL-18, and GSDMD are not specific markers of NLRP3; therefore, their alterations alone cannot establish NLRP3-specific inflammasome activation.

In addition to local inflammation and tissue damage, some clinical studies have suggested that inflammasome-related abnormalities are associated with disease severity and lymphoproliferative phenotypes. Vakrakou et al. found that patients with SjD exhibited elevated cell-free DNA (cfDNA), reduced DNase I activity, and heightened inflammasome responsiveness in circulating monocytes, with these abnormalities being more pronounced in patients with more severe disease or a higher risk of lymphoma (25). In addition, salivary gland abnormalities involving the P2X7 receptor (P2X7R)-NLRP3 inflammasome axis have been associated with lymphoid tissue organization and lymphoproliferative phenotypes (24). These findings suggest that inflammasome-related abnormalities are not restricted to local glands and the peripheral immune system in SjD, but may also be associated with disease severity and specific pathological phenotypes. However, the available human evidence remains primarily associative.

Genetic variation in *NLRP3* has also attracted attention. Li et al. analyzed polymorphisms in 285 patients with pSS and 170 healthy controls and found no significant association between the examined loci and overall susceptibility to pSS. However, some loci were associated with immunological phenotypes such as antinuclear antibody (ANA) positivity, suggesting that certain *NLRP3* loci may be related to immunological phenotypes in patients with pSS, although the available evidence is insufficient to establish a role for *NLRP3* genetic variation in disease susceptibility (31). Overall, current clinical evidence supports the presence of NLRP3 inflammasome-related abnormalities in the salivary glands, ocular surface, and peripheral immune system of patients with SjD. However, findings vary across studies with respect to tissue source, measured indicators, and changes in NLRP3 itself, and most evidence still comes from cross-sectional or observational studies. Therefore, these abnormalities are more appropriately regarded as a pathway associated with inflammation and glandular damage in SjD, while their specific roles across different patient populations and disease stages remain to be clarified.

### 4.2 Preclinical evidence

Preclinical studies further support the involvement of the NLRP3 inflammasome in SjD-related pathological processes. Current evidence is derived mainly from spontaneous or genetic SjD-like mouse models, antigen-induced experimental Sjögren syndrome (ESS) models, and cell models related to the salivary and lacrimal glands, which are often accompanied by changes such as caspase-1 activation, IL-1β and IL-18 release, and GSDMD-related pyroptosis (32–34). It should be noted that these models differ substantially in genetic background, disease stage, affected tissue, animal sex, and major pathological phenotype; therefore, their findings cannot be directly equated with those in human SjD. The major changes in the NLRP3 inflammasome and related effector molecules across different SjD-like mouse models are summarized in **Table 2**.

**Table 2:** NLRP3 inflammasome components and related molecules in mouse models of Sjögren disease.

Model	Comparator	Sex	Age / disease stage	Sample / tissue	Main SjD-like phenotype	Key molecular findings	Reference
NOD; spontaneous SjD-like model	Age-matched ICR mice	Female	8 weeks; SjD-like manifestations	SGs, lung, spleen, PBMCs, splenic MDSCs and plasma	Salivary flow ↓; SG enlargement and lymphocytic infiltration; lung injury	NLRP3: mRNA ↑ (SGs, spleen and PBMCs), protein ↑ (SGs, lung, spleen and MDSCs); ASC: mRNA ↑ (SGs, lung, spleen and PBMCs), protein ↑ (SGs, lung and spleen); caspase-1: pro-caspase-1 and p20 protein ↑ (SGs, lung, spleen and MDSCs); IL-1β: mRNA ↑ (SGs, spleen and MDSCs), plasma concentration ↑; IL-18: mRNA ↑ (SGs, lung and MDSCs), plasma concentration ↑	32
NOD; spontaneous SjD-like model	BALB/c mice	Female	Mainly 8 weeks; 12 weeks also reported	SMGs and serum	Salivary flow ↓; SMG enlargement and lymphocytic infiltration; serum ANA ↑	NLRP3: mRNA and protein ↑ (SMGs); ASC: mRNA and protein ↑ (SMGs); caspase-1: mRNA and protein ↑ (SMGs), cleaved form ↑; IL-1β: SMG homogenate and serum concentrations ↑; IL-18: SMG homogenate and serum concentrations ↑	33
NOD/ShiLtJ; spontaneous SjD-like model	Age-matched BALB/c mice	Female	Molecular assays: 12 weeks; IHC: 13–14 weeks	SMGs; serum for disease characterization	Salivary secretion ↓; SMG leukocyte infiltration; serum ANA positivity	NLRP3: protein NS (SMGs); IL-1β: level NS (SMGs); IL-18: level ↑ (SMGs)	35
NOD.B10Sn-H2^b^/J; spontaneous SjD-like model	Age-matched BALB/cJ mice	Male	RNA-seq: 2, 4 and 6 months; WB: 6 months	LGs	Progressive LG inflammation and lymphocytic infiltration with age	NLRP3: mRNA ↑ (LGs); caspase-1: mRNA ↑ (LGs), p33 protein ↑ and pro-caspase-1 NS at 6 months; IL-1β: mRNA ↑ (LGs), precursor and mature protein ↑ at 6 months; IL-18: mRNA ↑ (LGs)	34
C57BL/6.NOD-Aec1Aec2; spontaneous SjD-like model	Age-matched C57BL/6 mice	Female	14 weeks	SMGs and serum	Salivary secretion and SGI ↓; SMG injury, inflammatory infiltration and oxidative stress ↑	NLRP3: protein ↑ (SMGs); ASC: protein ↑ (SMGs); caspase-1: protein ↑ (SMGs); IL-1β: protein ↑ (SMGs, WB), concentration ↑ (SMGs and serum)	27 (USP5)
C57BL/6.NOD-Aec1Aec2; spontaneous SjD-like model	Age-matched C57BL/6 mice	Female	14 weeks	SMGs; cytokine sample source unspecified	Salivary secretion and SGI ↓; SMG lymphocytic infiltration, acinar damage and focal atrophy	NLRP3: protein ↑ (SMGs); IL-1β: concentration ↑; IL-18: concentration ↑	36 (GRP78)
C57BL/6.NOD-Aec1Aec2; spontaneous SjD-like model	Age-matched C57BL/6 mice	Female	14 weeks	SMGs and serum	Salivary secretion, tear secretion and SGI ↓; SMG acinar abnormalities, focal atrophy and lymphocytic infiltration	NLRP3: protein ↑ (SMGs); ASC: protein ↑ (SMGs); caspase-1: protein ↑ (SMGs); IL-1β: protein ↑ (SMGs, WB), serum concentration and SMG content ↑	37 (CaSR/rutin)
C57BL/6; SMG protein-induced ESS	Non-ESS C57BL/6 mice	Female	Initially 8 weeks; immunization on days 0, 7 and 14; sampling age unspecified	SMGs and serum	Salivary flow ↓; SMG lymphocytic foci and epithelial apoptosis ↑	NLRP3: staining and protein ↑ (SMGs); ASC: protein ↑ (SMGs); caspase-1: cleaved protein ↑ (SMGs); IL-1β: protein ↑ (SMGs, WB), serum concentration ↑; IL-18: protein ↑ (SMGs), serum concentration ↑	38

↑, increased relative to the normal comparator; ↓, decreased relative to the normal comparator; NS, no statistically significant difference; mRNA, protein and cytokine concentrations are reported separately; only normal–model comparisons are included.

ANA, antinuclear antibodies; ASC, apoptosis-associated speck-like protein containing a CARD; CARD, caspase recruitment domain; CaSR, calcium-sensing receptor; ESS, experimental Sjögren syndrome, as termed in the original study; GRP78, glucose-regulated protein 78; ICR, Institute of Cancer Research; IHC, immunohistochemistry; IL, interleukin; LGs, lacrimal glands; MDSCs, myeloid-derived suppressor cells; mRNA, messenger RNA; NLRP3, NLR family pyrin domain-containing 3; NOD, non-obese diabetic; PBMCs, peripheral blood mononuclear cells; RNA-seq, RNA sequencing; SGs, salivary glands; SGI, salivary gland index; SjD, Sjögren disease; SMGs, submandibular glands; USP5, ubiquitin-specific peptidase 5; WB, western blotting.

#### 4.2.1 Spontaneous/genetic SjD mouse models

Non-obese diabetic (NOD) mice and related strains are commonly used spontaneous animal models for studying SjD and can develop SjD-like manifestations with age, including lymphocytic infiltration of the salivary or lacrimal glands, tissue damage, and reduced secretory function. However, findings regarding NLRP3 inflammasome status have not been fully consistent across studies. Xia et al. observed reduced salivary secretion, glandular inflammation, and increased levels of NLRP3, ASC, pro-caspase-1, and activated caspase-1 in 8-week-old female NOD mice, accompanied by increased IL-1β and IL-18 (32). Jiang et al. obtained similar results in female NOD mice (33). In contrast, Zhou et al. found no significant difference in submandibular gland NLRP3 protein levels compared with controls in 11–12-week-old female NOD mice that had already developed SjD-like phenotypes, although IL-18 levels were increased (35). Notably, Xia et al. and Zhou et al. obtained opposite disease outcomes when NOD mice were treated with MCC950 (32, 35). However, these opposing outcomes cannot be attributed solely to age or disease stage, because the two studies also differed in baseline disease status, control strains, dosing regimens, and experimental design. These differences suggest that the effects of NLRP3 inhibition may be jointly shaped by disease stage, model background, and the local tissue environment.

Different NOD-related models further suggest that NLRP3 inflammasome abnormalities are dependent on the model, tissue, and sex. Delcroix et al. observed increased NLRP3 inflammasome- and pyroptosis-related molecules in the lacrimal glands of male NOD.H2^b^ mice, accompanied by enhanced activation of caspase-1 and GSDMD (34). In the C57BL/6.NOD-Aec1Aec2 model, Bulosan et al. found increased caspase-1 activity and abnormalities in IL-1β and IL-18 before the onset of overt glandular inflammation, although NLRP3 was not directly assessed (39). Subsequent studies observed increased levels of NLRP3, ASC, caspase-1, and IL-1β in this model (27). These findings suggest that inflammasome abnormalities in different NOD-related models may be influenced by age, sex, genetic background, and the affected gland, and therefore should not be directly compared across studies.

In addition to the above models, NOD.H-2h4 mice and their genetically modified strains have also been used in SjD research. Differences in genetic background can lead to variations in the timing of disease onset and the severity of glandular inflammation and secretory dysfunction (40, 41). Overall, animal studies directly evaluating the NLRP3 inflammasome remain concentrated in a limited number of NOD-related models, with considerable heterogeneity among their findings. This highlights the need to consider model background, disease stage, and target tissue when interpreting the role of NLRP3 in disease.

#### 4.2.2 Antigen-induced ESS model

In addition to spontaneous and genetic models, antigen-induced ESS models have also been used to study SjD-related inflammation. Existing studies directly evaluating the NLRP3 inflammasome have mainly focused on ESS models induced by salivary gland proteins. Zhang et al. found that ESS mice exhibited reduced salivary secretion, inflammatory cell infiltration and tissue damage in the submandibular glands, accompanied by increased levels of NLRP3 inflammasome-related molecules. Following iguratimod treatment, these phenotypes and related inflammatory indicators were improved (38). These findings suggest that NLRP3 inflammasome abnormalities are not restricted to NOD-background models but can also be observed in antigen-induced SjD-like models.

#### 4.2.3 Cell models

Cell-based studies have provided further mechanistic evidence supporting the involvement of the NLRP3 inflammasome in SjD-related inflammatory responses (**Table 3**). Peripheral blood mononuclear cells from patients can exhibit heightened inflammasome responsiveness (25), suggesting that circulating immune cells may contribute to inflammatory amplification in SjD; however, these findings cannot directly represent NLRP3 status in local glandular epithelial cells.

**Table 3:** NLRP3 inflammasome components and related molecules in Sjögren disease-related cell models.

Cell model	Species / source	Stimulus / condition	Comparator	Key molecular findings	Reference
Primary peripheral blood monocytes	Human; healthy-donor CD14^+^ monocytes	SjD serum cf-NA, 7 μg/mL, 16 h	Mock-treated vs SjD cf-NA-treated cells; healthy-donor cf-NA as an additional control	NLRP3: mRNA ↑; ASC: mRNA ↑; caspase-1: mRNA ↑; IL-1β: mRNA ↑	25
Primary SMG acinar cells	Mouse; normal female BALB/c mice	TNF-α, 10 ng/mL, 8 h	Untreated vs TNF-α-treated cells	NLRP3: protein ↑; caspase-1: protein ↑; IL-1β: protein ↑	42 (ruscogenin)
Primary mixed SMG cells	Mouse; NOD-derived epithelial cells and fibroblasts	LPS, 1 μg/mL, 3 h; then ATP, 5 mM, 30 min	Unstimulated vs LPS + ATP-treated cells	NLRP3: mRNA and protein ↑; ASC: mRNA and protein ↑; caspase-1: mRNA and protein ↑, cleaved caspase-1 ↑; IL-1β: supernatant concentration ↑; IL-18: supernatant concentration ↑	33
Primary splenic MDSCs	Mouse; 8-week-old female ICR and NOD mice	Ex vivo comparison of normal- and model-derived cells	ICR-derived vs NOD-derived cells	NLRP3: protein ↑; caspase-1: pro-caspase-1 and p20 protein ↑; IL-1β: mRNA ↑; IL-18: mRNA ↑	32
Primary SMG cells	Mouse; SMG-derived cells	LPS, 24 h; dose inconsistently reported	Untreated vs LPS-treated cells	NLRP3: protein ↑; caspase-1: protein ↑; IL-1β: assay level ↑; IL-18: assay level ↑	36 (GRP78)
Primary SMG cells	Mouse; SMG-derived cells	LPS, 50 μM as reported, 24 h	Untreated vs LPS-treated cells	NLRP3: protein ↑; ASC: protein ↑; caspase-1: protein ↑; IL-1β: protein ↑	37 (CaSR/rutin)
Primary SMG cells	Mouse; SMG-derived cells	LPS, 50 μM as reported, 24 h	Untreated vs LPS-treated cells	NLRP3: protein ↑; ASC: protein ↑; caspase-1: protein ↑; IL-1β: protein ↑	27 (USP5)
HSG cells	Human; purchased from iCell Bioscience	IFN-α, 10 ng/mL, 48 h	Untreated vs IFN-α-treated cells	NLRP3: protein and immunostaining ↑; ASC: protein ↑; caspase-1: cleaved caspase-1 protein ↑; IL-1β: cellular protein ↑; IL-18: cellular protein ↑	38
Primary SMG cells	Mouse; SMG-derived cells	LPS, 1 μg/mL, 3 h; then ATP, 5 mM, 30 min	Unstimulated vs LPS + ATP-treated cells	NLRP3: protein ↑; caspase-1: protein ↑, cleaved caspase-1 ↑; IL-1β: supernatant concentration ↑; IL-18: supernatant concentration ↑	43

↑, increased relative to the stated comparator; arrows indicate the direction of change and do not by themselves establish statistical significance; only control–model comparisons are included; mRNA expression, cellular protein levels and supernatant concentrations are recorded separately; precursor or cleaved forms are specified when identified in the original study.

ASC, apoptosis-associated speck-like protein containing a CARD; CARD, caspase recruitment domain; ATP, adenosine triphosphate; CaSR, calcium-sensing receptor; cf-NA, cell-free nucleic acids; GRP78, glucose-regulated protein 78; HSG, original human salivary/submandibular gland cell-line designation; ICR, Institute of Cancer Research; IFN, interferon; IL, interleukin; LPS, lipopolysaccharide; MDSCs, myeloid-derived suppressor cells; mRNA, messenger RNA; NLRP3, NLR family pyrin domain-containing 3; NOD, non-obese diabetic; SjD, Sjögren disease; SMG, submandibular gland; TNF, tumor necrosis factor; USP5, ubiquitin-specific peptidase 5.

Studies using primary murine salivary gland cells have shown that glandular epithelial cells are capable of mounting inflammasome responses, and ATP/P2X7R stimulation can induce NLRP3 inflammasome assembly and promote IL-1β release (22). In addition, TNF-α or LPS/ATP stimulation can enhance NLRP3-related inflammatory responses in primary submandibular gland cells (33, 42). These findings suggest that glandular epithelial cells are not merely targets of inflammatory injury but may also actively participate in local inflammatory responses.

In this context, the SjD-related inflammatory environment may further enhance the responsiveness of glandular epithelial cells to inflammasome stimulation. In the HSG (human salivary gland) cell line, pretreatment with IFN-α or IFN-β can enhance caspase-1/GSDMD-related pyroptosis and IL-18 release induced by subsequent inflammasome stimulation (26). These findings suggest that a type I interferon (IFN-I) environment may increase the responsiveness of glandular epithelial cells to inflammasome stimulation; however, substantial differences remain between HSG cells and primary salivary gland epithelial cells from patients.

In addition to glandular epithelial cells, NLRP3 inflammasome abnormalities in immune cells may also contribute to SjD-related inflammation. Xia et al. found enhanced NLRP3 inflammasome activation in spleen-derived myeloid-derived suppressor cells (MDSCs) from NOD mice, accompanied by increased mRNA expression of IL-1β and IL-18 (32), suggesting that myeloid immune cells may contribute to the maintenance of the SjD-like inflammatory microenvironment. In addition, primary lacrimal gland epithelial cells from aquaporin 5 (AQP5)-deficient mice also exhibited NLRP3-related inflammation and pyroptotic abnormalities (44). However, this model is not a classical autoimmune SjD model; therefore, its findings mainly provide supplementary evidence for mechanisms of lacrimal gland epithelial cell injury.

Overall, existing cell-based studies suggest that NLRP3 inflammasome abnormalities may occur in both glandular epithelial cells and immune cells and may contribute to inflammatory responses in SjD through IL-1β and IL-18 release and GSDMD-related pyroptosis. At the same time, the experimental systems used across studies differ substantially, including patient-derived immune cells, primary murine glandular cells, murine immune cells, and HSG cell lines, and their clinical relevance is therefore not equivalent. In particular, evidence directly using primary salivary gland epithelial cells from patients with SjD for functional validation of NLRP3 remains limited. Therefore, although existing cell-based studies provide important support for NLRP3-related mechanisms, further validation using patient glandular tissues and primary cells is still needed to establish disease specificity.

## 5 NLRP3 inflammasome and clinical phenotypes and systemic complications of SjD

In addition to salivary and lacrimal gland involvement, SjD can be accompanied by a variety of systemic manifestations and complications (3). Current evidence suggests that the NLRP3 inflammasome may participate in inflammatory processes associated with certain clinical phenotypes and complications. Relatively direct evidence supports its involvement in SjD-related dry eye and lymphoproliferative changes, whereas evidence regarding cardiovascular involvement and interstitial lung disease remains largely indirect and is based mainly on clinical associations and general mechanisms of NLRP3-mediated inflammation. This section discusses these clinical phenotypes and systemic manifestations according to the strength of the available evidence.

### 5.1 SjD-related dry eye and ocular surface damage

The development of SjD-related dry eye is closely associated with lacrimal gland inflammation, secretory dysfunction, and ocular surface injury. Existing studies suggest that the NLRP3 inflammasome may be involved in this process. Human studies have identified NLRP3 inflammasome-related abnormalities on the ocular surface of patients with SjD-related dry eye (23). Preclinical studies further suggest that inflammasome abnormalities and pyroptosis in lacrimal gland epithelial cells may contribute to ocular surface and lacrimal gland injury. Enhanced NLRP3-related pyroptosis has been observed in AQP5-deficient models (44), and inflammasome-related abnormalities have also been reported in NOD.H2^b^ and thrombospondin-1 (TSP-1)-deficient models (34). However, these models do not fully recapitulate human autoimmune SjD and are therefore more appropriately considered as supportive evidence for mechanisms of lacrimal gland injury.

### 5.2 SjD-related lymphoma

Patients with SjD have an increased risk of B-cell non-Hodgkin lymphoma, particularly mucosa-associated lymphoid tissue (MALT) lymphoma. Baldini et al. prospectively followed 147 patients with SjD and found that salivary gland expression of P2X7R, NLRP3, caspase-1, and IL-18 was associated with germinal center formation and showed more pronounced abnormalities in patients who subsequently developed MALT lymphoma (24). Vakrakou et al. also observed that inflammasome-related abnormalities were more prominent in patients at high risk of lymphoma or in those who had developed lymphoma (25). These findings support an association between NLRP3-related inflammatory abnormalities and lymphoid tissue organization and lymphoproliferative changes in SjD glands. However, they do not establish that NLRP3 directly drives lymphoma development or that it has independent predictive value.

### 5.3 SjD-related systemic involvement

Patients with SjD have an increased risk of cardiovascular disease and subclinical atherosclerosis. Systematic reviews and cohort studies have shown increased carotid intima-media thickness, a higher prevalence of atherosclerotic plaques, and an increased risk of cardiovascular events such as myocardial infarction and ischemic stroke (45, 46). In atherosclerosis more broadly, the NLRP3 inflammasome can be activated by danger signals such as cholesterol crystals and can promote vascular inflammation through IL-1β and IL-18 (47). On this basis, some studies have proposed that persistent inflammasome-related abnormalities may contribute to SjD-related cardiovascular injury (48, 49). However, direct experimental evidence demonstrating a role for NLRP3 in vascular tissues from patients with SjD or in SjD-specific animal models is still lacking. Therefore, this association remains largely an indirect inference based on general mechanisms of atherosclerosis.

Interstitial lung disease (ILD) is an important systemic manifestation of SjD. Anti-Ro52 antibody positivity is closely associated with an increased risk of ILD (50). In preclinical studies, Xia et al. observed NLRP3 inflammasome-related abnormalities in the lung tissues of NOD mice, suggesting that inflammasome activation may occur in the lungs in the context of SjD-like disease (32). However, pulmonary lesions in conventional NOD mice are characterized mainly by inflammatory infiltration and do not fully recapitulate the progressive interstitial fibrosis observed in human SjD-associated ILD. Although a recently established NOD/LtJ mouse model combined with bleomycin can reproduce pulmonary inflammation and fibrosis, the NLRP3 inflammasome has not yet been directly evaluated in this model (51). In addition, a recent review proposed that anti-Ro52-related immune responses may promote pulmonary inflammation and fibrosis through pathways involving NLRP3/IL-1β (52), but direct evidence from original experimental studies in SjD-associated ILD is currently lacking. Therefore, the involvement of NLRP3 in SjD-associated ILD should still be regarded as a mechanistic hypothesis requiring further validation.

## 6 Upstream regulatory mechanisms of the NLRP3 inflammasome in SjD

Existing studies suggest that NLRP3 inflammasome abnormalities may contribute to SjD-related inflammation and tissue damage, but the upstream regulatory mechanisms remain incompletely understood. General NLRP3 biology indicates that ATP, ion homeostasis disturbances, mitochondrial dysfunction, and endoplasmic reticulum stress can participate in inflammasome priming and activation (11, 16, 53). However, not all of these mechanisms have been directly validated in SjD. Therefore, this section focuses on currently relevant upstream regulatory pathways by integrating SjD-specific studies with mechanistic evidence from general NLRP3 biology. The major upstream regulatory factors, downstream effects, and potential pathological roles of the NLRP3 inflammasome in SjD are summarized in **Figure 1**.

**Figure 1:**
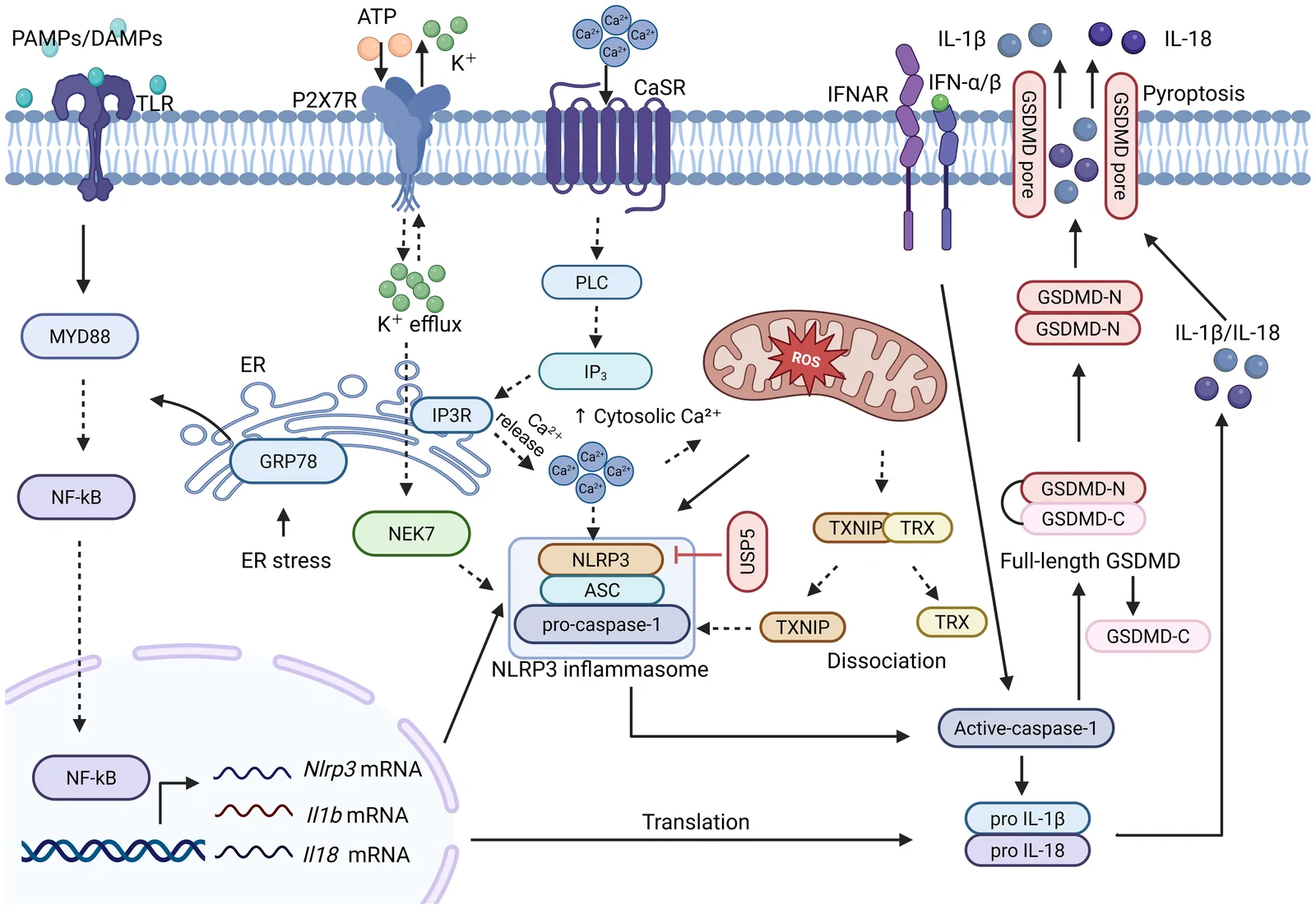
Molecular mechanisms regulating NLRP3 inflammasome activation and downstream effector responses in Sjögren disease (SjD). Priming of the NLRP3 inflammasome involves TLR/MyD88/NF-κB signaling and transcriptional regulation of NLRP3 and pro-IL-1β. Activation-related signals include extracellular ATP/P2X7R signaling, K⁺ efflux and NEK7, CaSR-mediated Ca^2+^ signaling, mitochondrial ROS, TXNIP/TRX dissociation, and endoplasmic reticulum stress/GRP78 signaling. USP5 negatively regulates NLRP3 through mechanisms related to NLRP3 ubiquitination and protein homeostasis. Following inflammasome assembly, caspase-1 activation promotes the maturation of IL-1β and IL-18 and cleavage of GSDMD, whose N-terminal fragment forms membrane pores to facilitate cytokine release and pyroptosis. In addition, type I interferons (IFN-α/β) acting through IFNAR can increase caspase-1 and GSDMD expression and enhance subsequent inflammasome-associated pyroptosis and IL-18 release in SjD-related epithelial cell models. Solid arrows indicate mechanistic relationships supported by functional perturbation evidence in SjD or SjD-related experimental systems, such as gene knockdown or deficiency, overexpression, pharmacological inhibition, or rescue experiments. Dashed arrows indicate relationships supported primarily by expression data, associations, or evidence of concurrent changes in SjD studies without direct functional validation, as well as mechanistic links inferred from broader NLRP3 biology. Created in BioRender. Rew, A. (2026) https://BioRender.com/vvncpb4.

### 6.1 TLR/NF-κB priming signal

Toll-like receptor (TLR)/nuclear factor-κB (NF-κB) signaling is one of the classical priming mechanisms of the NLRP3 inflammasome and can promote the expression of NLRP3 and pro-IL-1β, thereby providing the basis for subsequent inflammasome assembly and activation (11). In SjD-related studies, increased expression of multiple TLRs and myeloid differentiation primary response 88 (MyD88) has been observed in salivary gland epithelial cells and labial salivary gland tissues from patients, while TLR2 stimulation of patient PBMCs can also activate NF-κB-related inflammatory responses (54–56). Furthermore, *MyD88* deficiency in NOD.B10 mice can reduce exocrine gland and extraglandular inflammation and preserve salivary secretion, supporting the involvement of MyD88-dependent innate immune signaling in SjD-like disease (57). However, direct evidence that TLR/NF-κB-mediated NLRP3 priming plays a specific pathogenic role in SjD is still lacking; therefore, this mechanistic link remains largely based on general NLRP3 biology.

### 6.2 ATP/P2X7R signaling

ATP/P2X7R signaling is one of the classical activation pathways of the NLRP3 inflammasome. Binding of ATP to P2X7R can induce K^+^ efflux and promote the involvement of NEK7 in NLRP3 inflammasome assembly (58, 59).

The P2X7R-NLRP3 axis has relatively direct functional support in SjD-related models. Khalafalla et al. found that ATP stimulation promoted the assembly of NLRP3, ASC, and pro-caspase-1 in primary murine submandibular gland epithelial cells and induced IL-1β release, whereas P2X7R deficiency or NLRP3 inhibition significantly attenuated this response. N-acetylcysteine (NAC) and Mito-TEMPO, a mitochondrial reactive oxygen species (ROS) scavenger, also inhibited P2X7R-mediated IL-1β release, suggesting that ROS participates in this signaling process. In a SjD-like autoimmune exocrinopathy model, the P2X7R antagonist A438079 also reduced salivary gland inflammation and improved salivary secretion (22). Clinical studies further showed increased P2X7R expression and increased *NLRP3* and *CASP1* mRNA levels in labial salivary glands from patients with SjD, together with increased IL-18 protein levels in saliva; glandular *NLRP3* mRNA was positively correlated with focus score (20). In addition, increased P2X7R expression was observed in the lacrimal glands of NOD.H-2h4-related models and was associated with lacrimal gland inflammation (41). Therefore, existing cellular, animal, and human evidence supports the involvement of the P2X7R-NLRP3 axis in SjD-related glandular inflammation and secretory dysfunction at multiple levels.

### 6.3 Mitochondrial dysfunction and ROS

Mitochondrial dysfunction and associated oxidative stress may contribute to the upstream regulation of the NLRP3 inflammasome in SjD. Katsiougiannis et al. observed marked mitochondrial ultrastructural abnormalities in salivary gland epithelial cells from patients with SjD, accompanied by changes in metabolism and cellular bioenergetics (60). More recently, Carvajal et al. reported increased cytosolic release of mitochondrial DNA (mtDNA) in patient salivary gland epithelial cells, together with enhanced activation of innate immune sensing molecules, including cyclic GMP-AMP synthase (cGAS), Z-DNA-binding protein 1 (ZBP1), and NLRP3. Similar abnormalities were observed in the salivary glands of SjD mice and were partially reversed by tofacitinib (28). The P2X7R-mediated inflammasome response also shows ROS dependence (22), indicating that oxidative stress may connect upstream stimuli with inflammasome activation. In AQP5-deficient mice and lacrimal gland epithelial cells derived from these mice, Cao et al. observed ROS accumulation and increased levels of NLRP3, cleaved caspase-1, cleaved GSDMD, and cleaved IL-1β. Treatment with NAC or MCC950 attenuated these changes, supporting the involvement of ROS–NLRP3 signaling in lacrimal gland epithelial injury and pyroptosis (44). Although this is not a classical autoimmune SjD model, it provides supplementary evidence for mechanisms of glandular epithelial injury. In NOD mice and submandibular gland cells, activation of the nuclear factor erythroid 2-related factor 2 (Nrf2)/heme oxygenase-1 (HO-1) antioxidant pathway was associated with reduced NLRP3 inflammasome activation, whereas the Nrf2 inhibitor ML385 attenuated this effect (43). These observations support a role for mitochondrial abnormalities and oxidative stress in NLRP3-related inflammation, although the strength of disease-specific evidence varies among the experimental systems.

### 6.4 Ca²^+^ homeostasis and the calcium-sensing receptor

Abnormal Ca²^+^ homeostasis is an important feature of glandular dysfunction in SjD. Enger et al. found that intracellular Ca²^+^ responses to carbachol stimulation were significantly attenuated in freshly isolated salivary gland acinar cells from patients with SjD (61). In the IL14α SjD-like model, abnormalities in Ca²^+^ signaling were observed before the decline in salivary secretion and immune cell infiltration, and restoration of TRPC1-related Ca²^+^ homeostasis improved glandular function and inflammatory phenotypes (62). Huang et al. further observed dysregulated Ca²^+^ signaling in an early SjD-like model, accompanied by reduced salivary secretion and abnormalities in mitochondrial morphology and function (63). These studies indicate that disruption of Ca²^+^ homeostasis itself is an important functional abnormality in SjD glandular pathology.

In general NLRP3 biology, extracellular Ca²^+^ can promote NLRP3 inflammasome activation through pathways involving the calcium-sensing receptor (CaSR) (64). In SjD-related studies, He et al. identified increased CaSR expression in the submandibular glands of SjD mice by proteomic analysis and further demonstrated, using CaSR-overexpressing mice and primary submandibular gland cells, that CaSR overexpression could partially reverse the inhibitory effects of rutin on NLRP3, ASC, caspase-1, IL-1β, and p-NF-κB p65-related inflammatory signaling (37). These findings provide functional evidence for the involvement of CaSR in the regulation of the NLRP3 inflammasome in SjD and suggest that CaSR may represent an important regulatory node linking Ca²^+^ signaling to NLRP3 inflammasome activity.

### 6.5 Endoplasmic reticulum stress and GRP78

Endoplasmic reticulum stress may contribute to the dysregulation of the NLRP3 inflammasome in SjD. During endoplasmic reticulum stress, the unfolded protein response (UPR) is activated. Glucose-regulated protein 78 (GRP78, also known as BiP) is an important molecule involved in maintaining endoplasmic reticulum protein homeostasis and regulating UPR pathways, including PERK, IRE1, and ATF6. Coursey et al. observed increased *HSPA5* gene expression and GRP78 protein expression in conjunctival tissues from patients with SjD. In primary murine conjunctival goblet cells and related experimental dry eye models, they further found that IFN-γ induced endoplasmic reticulum stress and the UPR, accompanied by increased levels of GRP78, sXBP1, and other related molecules (65). Subsequently, Carvajal et al. observed abnormalities in integrated stress response (ISR)-related molecules, including PERK/p-PERK, eIF2α/p-eIF2α, and ATF4, in the labial salivary glands of patients with SjD (66). Cavalcanti et al. also observed abnormalities in UPR-related genes, including *EIF2AK3* (PERK), *XBP1*, and *HSPA5* (BiP), in minor salivary glands from patients, further supporting the presence of endoplasmic reticulum stress and UPR dysregulation in SjD glands (67). Regarding NLRP3-related mechanisms, He et al. found that GRP78 silencing reduced NLRP3 and caspase-1 expression and NF-κB p65 nuclear translocation, whereas GRP78 overexpression enhanced the related inflammatory response (36). These findings support GRP78 as an important regulatory node linking endoplasmic reticulum stress to NF-κB/NLRP3 inflammatory signaling.

### 6.6 Negative regulation by USP5

In addition to upstream signals that promote NLRP3 activation, negative regulation of NLRP3 protein homeostasis may also influence inflammasome responses in SjD. General mechanistic studies of NLRP3 have shown that ubiquitin-specific peptidase 5 (USP5) can act as a scaffold protein to recruit the E3 ubiquitin ligase MARCHF7, thereby promoting K48-linked polyubiquitination and autophagy-lysosomal degradation of NLRP3 and limiting excessive inflammasome activation. This effect is independent of the deubiquitinase activity of USP5 (68). In SjD-related studies, He et al. found decreased USP5 expression in the submandibular glands of C57BL/6.NOD-Aec1Aec2 mice. Knockdown of USP5 in primary submandibular gland cells enhanced NLRP3-dependent caspase-1 activation and IL-1β maturation and release, while having little effect on AIM2-, NLRC4-, or NF-κB-related signaling. Further immunoprecipitation experiments showed that USP5 could interact with NLRP3 and promote its ubiquitination (27). These findings suggest that USP5 can limit excessive NLRP3 activation by regulating its ubiquitination and protein stability, thereby contributing to the regulation of SjD-related inflammatory responses.

## 7 Downstream effector mechanisms of the NLRP3 inflammasome in SjD

The major upstream regulatory mechanisms of the NLRP3 inflammasome in SjD have been discussed above. Following inflammasome assembly, activated caspase-1 can cleave GSDMD and promote the maturation of pro-IL-1β and pro-IL-18, thereby mediating pyroptosis and the release of inflammatory mediators (11, 17). These processes constitute the major downstream effects of the NLRP3 inflammasome. However, abnormalities in GSDMD, IL-1β, and IL-18 do not necessarily indicate NLRP3-specific activation. Therefore, this section evaluates disease-specific evidence for GSDMD-mediated pyroptosis and IL-1β- and IL-18-related effects based on studies involving patients with SjD, animal models, and cell experiments.

### 7.1 GSDMD and pyroptosis

GSDMD is a key effector molecule of pyroptosis mediated by the canonical inflammasome/caspase-1 pathway and is also an important downstream effector of the NLRP3 inflammasome. Following activation of the canonical NLRP3 inflammasome, caspase-1 cleaves GSDMD and releases its pore-forming N-terminal fragment, which forms pores in the cell membrane, leading to cell swelling, disruption of membrane integrity, and the release of mature IL-1β and IL-18, thereby amplifying local inflammatory responses (17, 18).

SjD-related studies suggest that pyroptosis of glandular epithelial cells may be regulated by the local inflammatory environment. Hong et al., using patient salivary gland tissues and HSG cells, found that type I interferons enhanced caspase-1/GSDMD-related pyroptosis and IL-18 release in glandular epithelial cells, supporting a functional link between the IFN-I inflammatory environment and inflammasome-related pyroptosis (26). Delcroix et al. further observed enhanced cleavage of caspase-1 and GSDMD in the lacrimal glands of NOD.H2^b^ mice (34). These findings suggest that GSDMD-mediated pyroptosis may represent an important effector mechanism of inflammasome-related glandular injury, although its upstream regulation is not necessarily restricted to NLRP3. Functional intervention studies further showed that inhibition of NLRP3 reduced GSDMD activation and pyroptosis in lacrimal gland epithelial cells (44), supporting a functional link between NLRP3 and pyroptosis. In addition, Yu et al. observed pyroptosis-related changes in KRT14^+^ cells in the labial salivary glands of patients with pSS and found increased cleaved GSDMD and reduced salivary secretory function in *Atg5* conditional knockout mice, whereas inhibition of pyroptosis partially restored salivary flow (69). These studies suggest that GSDMD-mediated pyroptosis may contribute to SjD-related glandular epithelial cell injury and secretory dysfunction and may represent an important mechanism of NLRP3 inflammasome-mediated tissue injury.

### 7.2 IL-1β

IL-1β is an important downstream pro-inflammatory cytokine of the canonical inflammasome/caspase-1 pathway. Following NLRP3 inflammasome activation, caspase-1 cleaves pro-IL-1β into mature IL-1β, which can be released through pores formed by GSDMD and contribute to local inflammation and tissue damage. However, IL-1β maturation and release are not specific to NLRP3; increased IL-1β expression or concentration alone therefore cannot establish NLRP3 inflammasome activation. In patients with SjD, increased *IL1B* mRNA expression and mature IL-1β protein have been reported in PBMCs, with *IL1B* mRNA expression associated with disease-related damage (21).

IL-1 signaling itself may also directly contribute to SjD-related tissue damage. Chen et al. observed increased IL-1β expression on the ocular surface in an *Aire*-deficient SjD-like dry eye model, whereas deficiency of interleukin-1 receptor type 1 (IL-1R1) significantly reduced ocular surface keratinization, epithelial damage, and mucin abnormalities. Further adoptive transfer experiments showed that local tissue IL-1R1 signaling promoted CD4^+^ T-cell retention and persistent ocular surface inflammation (70, 71). These findings provide functional evidence for the involvement of IL-1 signaling in SjD-related ocular surface injury. Notably, in NOD mice with established SjD-like lesions, IL-18 levels decreased significantly following NLRP3 inhibition with MCC950, whereas IL-1β levels were low and did not change significantly (35), suggesting that the source of IL-1β and its dependence on NLRP3 activation may vary across disease stages and tissue environments. Overall, current evidence supports IL-1β as an important downstream effector of the NLRP3 inflammasome and indicates that IL-1 signaling can promote SjD-related tissue inflammation and damage.

### 7.3 IL-18

IL-18 is an important downstream cytokine of the inflammasome/caspase-1 pathway. Pro-IL-18 is cleaved by caspase-1 to generate mature IL-18, which mediates subsequent immune responses through the IL-18 receptor (IL-18R). Relatively consistent human evidence indicates dysregulation of IL-18 in patients with SjD. Bombardieri et al. found increased serum IL-18 levels in patients with pSS, together with enhanced IL-18 expression in inflammatory infiltrates and ductal epithelial cells of the salivary glands. Serum IL-18 levels were also correlated with anti-SSA/Ro and anti-SSB/La antibody levels (72). Further studies showed that serum levels of total IL-18 and IL-18-binding protein (IL-18BP) were increased in patients with pSS, and calculated free IL-18 levels were also elevated (73). Because IL-18BP is a natural high-affinity inhibitor of IL-18 and free IL-18 more closely reflects biologically active IL-18, these findings suggest that the compensatory increase in IL-18BP does not fully offset the increased IL-18 activity in patients with SjD. More recently, Parisis et al. further found increased serum levels of IL-18 and IL-18BP in patients with pSS, together with enhanced expression of IL-18, IL-18BP, and IL-18Rα in salivary ducts and inflammatory infiltrates, further supporting the involvement of the IL-18/IL-18BP/IL-18R axis in local immune inflammation in SjD (30).

Regarding NLRP3-related mechanisms, Baldini et al. found increased P2X7R expression and increased *NLRP3* and *CASP1* mRNA levels in labial salivary glands from patients with SjD, accompanied by increased IL-18 protein levels in saliva (20). In NOD mice, mature IL-18 increased with the onset and progression of SjD-like disease and was predominantly localized to the ductal regions of the submandibular glands. Following treatment with the NLRP3 inhibitor MCC950, IL-18 levels in the submandibular glands were significantly reduced, providing further functional evidence that IL-18 is regulated by the NLRP3 inflammasome (35). Notably, the reduction in IL-18 in this study was not accompanied by disease improvement; instead, salivary secretion decreased and glandular inflammation increased, suggesting that the biological effects of the NLRP3–IL-18 axis in SjD may depend on disease stage and the immune environment. Overall, existing evidence supports IL-18 as an important downstream effector of the NLRP3 inflammasome in SjD, although its net immunological effect during disease progression cannot be regarded as simply pro-inflammatory.

## 8 Intervention strategies targeting the NLRP3 inflammasome and related pathways in SjD

Existing studies suggest that the NLRP3 inflammasome and its related signaling pathways may represent potential therapeutic targets in SjD; however, the strength of evidence linking different therapeutic approaches to NLRP3 varies considerably. Currently, studies directly targeting NLRP3 in SjD are largely limited to preclinical investigations using agents such as MCC950, whereas some immunomodulatory drugs and naturally derived interventions with clinical research support are not NLRP3-specific therapies, and their associations with NLRP3 are based mainly on mechanistic studies. Therefore, this section discusses clinical interventions, direct NLRP3 inhibition, and preclinical multitarget interventions according to the level of evidence, with the aim of more accurately evaluating their potential therapeutic value and prospects for clinical translation.

### 8.1 Clinical evidence

At present, interventions targeting NLRP3 inflammasome-related pathways that have been investigated clinically in SjD mainly include iguratimod, total glucosides of paeony (TGP), and IL-1 receptor blockade; however, none of these interventions is an NLRP3-specific inhibitor.

Iguratimod (IGU) has shown therapeutic activity in previous clinical studies of pSS. A randomized, placebo-controlled trial conducted by Shao et al. included 66 patients with pSS and showed that 24 weeks of IGU treatment improved the EULAR Sjögren’s Syndrome Patient Reported Index (ESSPRI) and had favorable effects on the Schirmer test, erythrocyte sedimentation rate (ESR), and immunoglobulin G (IgG) levels (74). More recently, a multicenter randomized controlled trial involving 78 patients with active pSS compared the efficacy of IGU with hydroxychloroquine over 24 weeks. Although the primary endpoint, the Sjögren’s Syndrome Responder Index-30 (SSRI-30), was not significantly better in the IGU group than in the hydroxychloroquine group, secondary endpoints, including the Sjögren’s Tool for Assessing Response (STAR) and EULAR Sjögren’s Syndrome Disease Activity Index (ESSDAI) response rates and reductions in IgG levels, showed more favorable results (75). Meanwhile, preclinical studies have shown that IGU can reduce NLRP3 inflammasome-related activation in ESS mice and IFN-α-stimulated HSG cells (38). Therefore, current evidence supports the clinical therapeutic activity of IGU in SjD and suggests that inhibition of the NLRP3 inflammasome may contribute to its anti-inflammatory effects. However, the actions of IGU also involve B cells, T follicular helper (Tfh) cells, and other immunoregulatory pathways.

Total glucosides of paeony (TGP) also have a relatively well-supported clinical evidence base. A multicenter, randomized, double-blind, placebo-controlled trial involving 320 patients with pSS showed that the improvement in ESSPRI after 24 weeks of TGP treatment was significantly greater than that with placebo, with additional improvements in some sicca symptoms, fatigue, the Schirmer test, and ESR (76). Similar clinical and immunomodulatory effects were also observed in an earlier small-sample randomized controlled trial (77). Subsequent SjD animal studies showed that TGP treatment was accompanied by improved salivary secretion, reduced glandular inflammation, and decreased levels of NLRP3 inflammasome-related molecules (33). Therefore, the clinical efficacy of TGP is supported by randomized controlled trials, whereas its association with the NLRP3 inflammasome is currently based mainly on preclinical mechanistic studies.

Clinical evidence for interventions targeting the IL-1 pathway downstream of NLRP3 remains relatively limited. Norheim et al. conducted a double-blind, randomized, placebo-controlled trial in 26 patients with pSS using the IL-1 receptor antagonist anakinra for 4 weeks. The primary endpoint showed no significant improvement in fatigue scores in the treatment group compared with the placebo group. Although a higher proportion of patients achieved a 50% reduction in fatigue visual analog scale scores in a *post hoc* analysis, this finding cannot substitute for the prespecified primary endpoint (78). Thus, this study did not demonstrate a significant improvement in fatigue with anakinra in patients with pSS based on the prespecified primary endpoint; its effects on glandular inflammation, secretory function, and overall disease activity require further dedicated evaluation.

### 8.2 Direct NLRP3 inhibition

MCC950 is currently the most widely used selective NLRP3 inflammasome inhibitor in SjD research. It can directly bind to a region near the Walker B motif within the NACHT domain, inhibit ATP hydrolysis, and restrict NLRP3 conformational changes and inflammasome assembly, thereby reducing caspase-1 activation and the maturation and release of downstream IL-1β and IL-18 (79, 80). Because its molecular target is relatively well defined, MCC950 also serves as an important pharmacological tool for evaluating the function of NLRP3 under different disease conditions in SjD.

However, the effects of MCC950 intervention in NOD mice have not been consistent. Differences between the two studies in model status, MCC950 dosing regimens, and major outcomes are summarized in **Table 4**. Xia et al. administered MCC950 at 10 mg/kg to female NOD mice beginning at 8 weeks of age by intraperitoneal injection three times per week for 4 weeks. They observed reductions in NLRP3, caspase-1 p20, ASC, and other inflammasome-related markers in salivary gland and lung tissues, together with decreased serum IL-1β and IL-18 levels, improved salivary secretion, and reduced lymphocytic infiltration in the salivary glands and lungs. NLRP3 inflammasome activation in MDSCs was also inhibited (32). These findings support the conclusion that NLRP3 inhibition can reduce glandular inflammation and improve secretory function under these specific model and intervention conditions. In contrast, Zhou et al. administered MCC950 at 0.2 mg/mouse by intraperitoneal injection three times per week to 11-week-old female NOD mice that had already developed reduced salivary secretion and SjD-like pathological changes. After 1 week of treatment, IL-18 levels in the submandibular glands decreased; however, after 3 weeks of continuous treatment, salivary secretion was further reduced and glandular leukocyte infiltration increased, accompanied by increases in CD4^+^ and CD8^+^T cells, B cells, and Th1 cells, as well as reduced AQP5 expression (35). These findings suggest that, once SjD-like lesions are established, sustained NLRP3 inhibition does not produce the same protective effects observed by Xia et al. and may instead disrupt local immune homeostasis.

**Table 4:** Comparison of MCC950 treatment protocols and outcomes in Sjögren disease-like NOD mice.

Comparison item	Xia et al., 2023 (32)	Zhou et al., 2023 (35)
Mouse model and sex	Female NOD mice; substrain unspecified	Female NOD/ShiLtJ mice
Animal source	Nanjing University Model Animal Research Center	Jackson Laboratory
Age at treatment initiation	8 weeks	11 weeks
Disease manifestations at baseline	Hyposalivation; SG enlargement and lymphocytic infiltration; lung injury	Established hyposalivation; serum ANA positivity
Comparators	NOD mice without MCC950; age-matched ICR mice for baseline comparisons	PBS–DMSO-treated NOD mice; BALB/c mice for baseline salivary function
MCC950 dose	10 mg/kg per injection	0.2 mg/mouse per injection
Route and frequency	i.p.; three times/week	i.p.; three times/week
Treatment duration and sampling	4 weeks; approximately 12 weeks old at completion	3 weeks for disease outcomes; separate 1-week cohort for glandular molecular assays; sampling 1–2 days after the final injection
Samples	SGs, lung, splenic MDSCs and serum	SMGs, draining lymph nodes and serum
Main assays	Histology, WB, flow cytometry and ELISA	Histology, flow cytometry, IHC and ANA immunofluorescence
Salivary function	Salivary flow ↑	Salivary flow ↓
Histopathological changes	Lymphocytic infiltration ↓ (SGs and lung)	Leukocyte infiltration and lymphocytic focus burden ↑ (SMGs)
NLRP3 and related molecular findings	NLRP3: protein ↓ (SGs and lung); ASC: protein ↓ (SGs and lung); caspase-1: pro-caspase-1 and p20 protein ↓ (SGs and lung); IL-1β: serum concentration ↓; IL-18: serum concentration ↓; inflammasome activation markers ↓ (MDSCs)	At 1 week: NLRP3 staining NS (SMGs); IL-1β staining NS (SMGs); IL-18 staining ↓ (SMGs)
Additional findings	Splenic MDSC proportion ↓	At 3 weeks: T-cell, B-cell, Th1 and Treg proportions ↑ (SMGs); Th17 proportion NS; AQP5 ↓; serum ANA levels NS
Overall disease outcome	Improved glandular function and reduced inflammation	Worsened glandular function and increased inflammation

↑, increased relative to the respective model control; ↓, decreased relative to the respective model control; NS, no statistically significant difference; treatment outcomes describe changes following MCC950 administration.

ANA, antinuclear antibodies; AQP5, aquaporin 5; ASC, apoptosis-associated speck-like protein containing a CARD; CARD, caspase recruitment domain; DMSO, dimethyl sulfoxide; ELISA, enzyme-linked immunosorbent assay; ICR, Institute of Cancer Research; IHC, immunohistochemistry; IL, interleukin; i.p., intraperitoneal; MDSCs, myeloid-derived suppressor cells; NLRP3, NLR family pyrin domain-containing 3; NOD, non-obese diabetic; PBS, phosphate-buffered saline; SGs, salivary glands; SjD, Sjögren disease; SMGs, submandibular glands; Th1, T helper 1 cells; Th17, T helper 17 cells; Treg, regulatory T cells; WB, western blotting.

The discrepancy between these two studies cannot simply be attributed to the idea that “early inhibition is beneficial whereas late inhibition is harmful.” In addition to differences in the age at treatment initiation and baseline disease status, the studies also differed in the way MCC950 dose was expressed, treatment duration, control strains, and outcome measures. Therefore, it remains uncertain whether disease stage is the sole or principal factor responsible for the opposing results. A more reasonable interpretation is that the function of NLRP3 in SjD may be influenced by multiple factors, including disease status, the local tissue environment, and experimental design. This also suggests that appropriate intervention windows and disease subtypes may need to be considered when using direct NLRP3 inhibitors.

Taken together, current studies indicate that the effects of direct NLRP3 inhibition in preclinical SjD models are clearly context dependent. At present, these findings are insufficient to define a uniform therapeutic window, and the results of MCC950 animal studies cannot be directly extrapolated to patients. Future studies should perform standardized comparisons across different disease stages and model backgrounds and further integrate target-tissue findings, immune phenotypes, and long-term intervention outcomes to identify the disease stages and patient subgroups most likely to benefit from NLRP3 inhibition.

### 8.3 Preclinical multitarget interventions and natural compounds

Various naturally derived interventions and their active constituents have been shown to improve glandular inflammation and secretory function in SjD animal models or cell systems, accompanied by reductions in NLRP3 inflammasome-related signaling. However, these compounds generally exert multitarget effects, and their therapeutic effects cannot be simply attributed to NLRP3 inhibition. Therefore, based on the strength of mechanistic evidence, this section distinguishes interventions that are associated only with reductions in NLRP3-related markers from studies that include functional validation through knockdown, overexpression, or pathway blockade, thereby allowing a more accurate assessment of the relationship between different natural compounds and NLRP3 inflammasome regulation.

The clinical efficacy of total glucosides of paeony (TGP) has received some support from the randomized controlled trials described above, whereas its association with the NLRP3 inflammasome is based mainly on preclinical studies. Jiang et al. administered TGP orally at 400 mg/kg daily for 4 weeks in NOD mice and observed improved salivary secretion, reduced lymphocytic infiltration in the submandibular glands, and decreases in NLRP3 inflammasome-related molecules, IL-1β, and IL-18. TGP at 100 μg/mL also inhibited NLRP3 inflammasome activation in LPS- and ATP-stimulated submandibular gland cells (33). These findings associate the protective effects of TGP with NLRP3 regulation, but do not establish that these effects depend on NLRP3 because target-specific knockdown or rescue experiments were not performed.

Paeoniflorin (PF) is one of the major active components of TGP. Jiang et al. administered PF orally at 50 and 100 mg/kg daily for 30 days in NOD mice and observed alleviation of submandibular gland injury and reductions in NLRP3 inflammasome-related markers. In LPS- and ATP-stimulated submandibular gland cells, PF at 50–150 μg/mL activated the Nrf2/HO-1 pathway and inhibited NLRP3 inflammasome activation. The Nrf2 inhibitor ML385 attenuated PF-induced suppression of NLRP3, supporting the involvement of Nrf2/HO-1 signaling in its anti-inflammatory effects (43).

Forsythiaside A (FA) has been linked to endoplasmic reticulum stress and NLRP3 inflammasome regulation in SjD-related models. Oral FA at 20 mg/kg for 8 weeks improved salivary secretion and submandibular gland pathology in C57BL/6.NOD-Aec1Aec2 mice, accompanied by decreased glandular GRP78 and NLRP3 expression. In LPS-stimulated primary submandibular gland cells, FA at 6 and 30 μM reduced NLRP3, caspase-1, and nuclear p65 levels. GRP78 silencing reduced inflammasome-related signaling and inflammatory cytokine release, whereas GRP78 overexpression enhanced these responses. These findings support the involvement of GRP78 in FA-associated regulation of NF-κB/NLRP3 signaling (36).

The effects of salvianolic acid B (SaB) have been associated with USP5/NLRP3 regulation. In C57BL/6.NOD-Aec1Aec2 mice, oral SaB at 50 mg/kg for 8 weeks restored the reduced USP5 levels and alleviated oxidative stress and glandular inflammation, accompanied by reductions in NLRP3 inflammasome-related molecules. In LPS-stimulated primary submandibular gland cells, SaB at 5 μM also increased USP5 and reduced NLRP3-related molecules. Functional experiments showed that USP5 interacts with NLRP3 and increases its polyubiquitination, supporting USP5-mediated negative regulation of NLRP3 as a possible mechanism of SaB action (27).

The effects of rutin in SjD-related models have been associated with regulation of CaSR/NLRP3/NF-κB signaling. He et al. identified increased CaSR expression in the submandibular glands of SjD mice and used oral rutin at 50 mg/kg for 8 weeks in C57BL/6.NOD-Aec1Aec2 mice and 5 μM in primary submandibular gland cell experiments. Rutin improved salivary secretion, alleviated oxidative stress and inflammatory responses, and reduced NLRP3 inflammasome-related signaling. CaSR overexpression partially counteracted these effects, providing functional support for the involvement of CaSR in its anti-inflammatory activity (37).

In studies of ruscogenin in NOD/ShiLtJ mice, oral doses of 0.3 and 1 mg/kg were administered daily for 9 weeks, with the 1 mg/kg dose generally producing more pronounced effects. *In vitro* experiments using primary acinar cells employed concentrations of 0.1, 1, and 10 μM. Ruscogenin reduced submandibular gland inflammation and reduced the levels of P2X7R, NLRP3, caspase-1, and IL-1β. NLRP3 overexpression partially reversed its inhibitory effects on inflammation and cellular injury (42). These findings provide functional evidence for the involvement of NLRP3 in the protective effects of ruscogenin. However, the currently reported effective doses remain limited to animal and *in vitro* experiments, and human pharmacokinetic and target-tissue exposure data are still lacking.

Overall, these interventions provide preclinical evidence linking natural compounds to NLRP3-related regulation, with functional support in some studies. Studies of FA, SaB, rutin, and ruscogenin were conducted by the same or highly overlapping research groups using similar experimental systems, and independent replication was not identified among the studies reviewed. These compounds have multiple pharmacological actions, and the studies do not establish them as selective NLRP3 inhibitors. Whether the effective animal doses and cell concentrations translate into achievable human exposure in target tissues remains unclear. Pharmacokinetics, bioavailability, toxicity, and long-term safety require further evaluation. Clinical evidence in SjD is available for TGP, whereas the other compounds discussed here remain supported mainly by preclinical studies.

## 9 Discussion and future perspectives

Taken together, current evidence suggests that the NLRP3 inflammasome in SjD is more likely to function as an inflammatory amplification and regulatory node in the context of pre-existing autoimmunity and tissue stress, rather than as a universal independent initiating driver across all patients and disease stages. Abnormalities in NLRP3 inflammasome components, IL-1β, IL-18, and pyroptosis-related molecules have been observed in the salivary glands, ocular surface, and peripheral immune cells of patients, with some of these abnormalities also being associated with the severity of glandular inflammation and specific disease phenotypes. More importantly, P2X7R deficiency or antagonism, pharmacological inhibition of NLRP3, and related functional interventions can alter inflammasome activation, glandular inflammation, and secretory function, indicating that NLRP3 is not merely associated with inflammation but can actively participate in SjD-like pathological processes. At the same time, SjD glands exhibit broader epithelial stress and innate immune abnormalities, including sustained activation of IFN/JAK/STAT signaling (81), as well as mitochondrial dysfunction, cytosolic mtDNA release, and enhanced danger-sensing pathways involving cGAS, ZBP1, and NLRP3 (28, 60). These findings suggest that NLRP3 is more likely to be embedded within pre-existing networks of immune activation and tissue stress, where it further amplifies or modulates local inflammation. However, this interpretation does not exclude the possibility that NLRP3 may act more like a disease driver in certain patient subgroups or at specific disease stages. Human studies remain predominantly cross-sectional and associative, and changes in NLRP3 itself are not fully consistent across tissues and studies. Therefore, the precise role of NLRP3 over the course of SjD still requires careful definition.

This context dependence is also evident across different cell populations. Current evidence indicates that both glandular epithelial cells and myeloid cells can exhibit inflammasome-related abnormalities, although their functional roles in disease may differ. Glandular epithelial cells can respond to P2X7R signaling, type I interferons, oxidative stress, and mitochondrial danger signals, and their NLRP3-related responses may contribute more directly to epithelial injury, pyroptosis, and secretory dysfunction. In contrast, abnormalities in circulating monocytes, macrophages, and MDSCs may be more closely involved in inflammatory amplification and immune regulation (22, 25, 26, 28, 32). Therefore, the functions of NLRP3 across different cell types should not be regarded as equivalent, and its net effects may depend on the target cell, local inflammatory environment, and disease stage. However, current studies still lack cell-specific intervention evidence that directly compares the relative contributions of different cellular sources.

In addition to cellular origin, disease stage and the local microenvironment may further influence the net effects of NLRP3 in SjD, and the contrasting outcomes of MCC950 intervention provide an important clue in this regard. Xia et al. initiated MCC950 treatment in NOD mice at 8 weeks of age and observed improved salivary secretion, reduced glandular inflammation, and decreased IL-1β and IL-18 levels. In contrast, Zhou et al. inhibited NLRP3 in 11-week-old NOD mice with established SjD-like phenotypes and found that, although IL-18 levels in the submandibular glands were significantly reduced, salivary secretion declined further, while glandular inflammation and infiltration by multiple lymphocyte populations were exacerbated (32, 35). These contrasting findings indicate that NLRP3 inhibition does not necessarily produce a uniform protective effect and that its effects may be jointly influenced by disease status and the tissue microenvironment. It should be noted that the two studies differed in the timing of intervention, control strains, baseline pathological severity, and experimental conditions. Therefore, based on these two studies alone, the effects of NLRP3 inhibition cannot be simply summarized as “beneficial at an early stage and harmful at a later stage.” One possible explanation is that the balance among inflammatory responses, immune regulation, and tissue repair differs across disease states, resulting in different net effects of NLRP3 inhibition. This interpretation is also consistent with the broader understanding that NLRP3 exerts cell type- and context-dependent functions (19). However, current studies cannot distinguish the respective contributions of disease stage, model background, and experimental conditions, nor can they determine whether the adverse outcomes result from disruption of local immune homeostasis. These possibilities require further validation through stage-specific interventions within the same model and cell-specific studies.

From a histopathological perspective, existing human studies generally suggest that NLRP3-related abnormalities become more pronounced with increasing glandular inflammation, more organized lymphocytic infiltrates, and more prominent lymphoproliferative phenotypes (20, 24, 25, 72). This consistent trend supports the possibility that NLRP3-related inflammation contributes to a persistent pathological environment within the glands. However, current evidence remains insufficient to establish a continuous causal chain from NLRP3 activation to germinal center formation and ultimately to lymphoma development. Therefore, these abnormalities are currently more appropriately regarded as associative features reflecting pathological status rather than established pathogenic drivers or independent predictive markers.

The clinical and histological associations described above suggest that NLRP3-related markers may have potential value as candidate biomarkers. Across different studies, abnormalities in NLRP3 inflammasome components, IL-1β, IL-18, P2X7R, and cfDNA have been observed in salivary glands, peripheral blood, serum, saliva, and tears from patients with SjD. Some of these markers are associated with the severity of glandular inflammation, focus score, germinal center formation, or lymphoma development, suggesting that they may help identify patients with more pronounced inflammation or specific pathological phenotypes (20, 24, 25). Among these markers, IL-18-related abnormalities have been observed in multiple human studies. More recent studies have also identified increased IL-18BP levels in the serum and salivary glands of patients with SjD, providing a basis for further investigation of the biomarker potential of IL-18 and its regulatory network (21, 30). However, these biological associations are insufficient to establish their clinical utility. In addition, the clinical significance of candidate molecular biomarkers needs to be evaluated within existing clinical, serological, and histopathological diagnostic frameworks. A recent systematic review of diagnostic approaches for pediatric SjD showed that, given the substantial heterogeneity of clinical manifestations and the lack of pediatric-specific classification criteria, minor salivary gland biopsy remains an important diagnostic tool and should be interpreted in conjunction with clinical manifestations and serological findings (82). Although this study did not directly address NLRP3, it highlights that the clinical value of novel molecular biomarkers needs to be validated by comparison with established clinical and histopathological measures. On the one hand, abnormalities in the markers described above do not specifically reflect NLRP3 inflammasome activation and may also be influenced by sample source, disease stage, and other inflammatory pathways. On the other hand, their ability to support patient stratification, disease monitoring, or risk prediction has not yet been sufficiently validated in independent cohorts. Therefore, these markers are currently more appropriately regarded as candidate biomarkers requiring further validation, and their incremental value beyond existing clinical and pathological measures remains to be established.

From the perspective of clinical translation, the functional intervention studies described above support the targetability of the NLRP3 inflammasome and its related regulatory networks, but their clinical efficacy and safety in SjD remain to be established. The potential hepatotoxicity encountered during the previous clinical development of MCC950 highlights that the pathological relevance and targetability of a molecular target cannot substitute for evaluation of the safety of a specific drug (83). In recent years, direct NLRP3 inhibitors have entered early-stage clinical studies in gout, COVID-19 pneumonia, and ulcerative colitis (84–86), and their anti-inflammatory effects have also been investigated in populations at increased cardiovascular risk (87). These studies support the clinical druggability of NLRP3; however, findings from early-stage studies in other diseases cannot be directly extrapolated to SjD. At the time of the review by Cabral et al. in 2025, no NLRP3-targeted inhibitor had been approved by the U.S. Food and Drug Administration (FDA) (88). In addition to direct inhibition strategies, multitarget interventions such as IGU, TGP, PF, FA, SaB, rutin, and ruscogenin have also been associated with reductions in NLRP3-related signaling in SjD-related studies, and some studies have provided functional support through pathway inhibition, gene silencing, or overexpression. However, the simultaneous improvement of disease phenotypes and reduction of NLRP3-related markers is insufficient to demonstrate that the therapeutic effects are dependent on NLRP3. Target-specific interventions and rescue experiments are still required to clarify the contribution of NLRP3 to the overall therapeutic effect. Clinical findings for interventions targeting downstream pathways likewise need to be interpreted in the context of specific endpoints. In a randomized, double-blind, placebo-controlled trial in patients with pSS, the IL-1 receptor antagonist anakinra did not show a significant benefit in the prespecified primary endpoint of fatigue score after 4 weeks of treatment (78). This finding primarily reflects the limited evidence for IL-1 pathway blockade in improving fatigue, whereas its effects on glandular inflammation, secretory function, and overall disease activity require dedicated evaluation. Therefore, further development of NLRP3-related therapeutic strategies in SjD will require identification of appropriate intervention stages and patient populations on the basis of clearly established target dependency and drug safety, together with clinical endpoints aligned with the expected therapeutic effects.

Overall, the NLRP3 inflammasome can be regarded as an important regulatory node linking innate immune abnormalities, glandular epithelial injury, inflammatory amplification, and local immune remodeling in SjD, although its specific role may vary according to cell type, disease status, and the local microenvironment. Future research should shift from continuing to establish whether NLRP3 is abnormal toward determining in which cell types, at which disease stages, and in which patient populations it has genuine pathological and therapeutic significance. Mechanistic studies should increasingly incorporate patient-derived primary cells, single-cell and spatial omics approaches, and cell-specific genetic models to clarify the relative contributions and interactions of NLRP3 in epithelial and myeloid cells. Clinical studies should establish longitudinal, multicenter cohorts with independent validation cohorts and systematically integrate NLRP3-related markers with glandular pathology, disease activity, lymphoma risk, and treatment response, while further evaluating their discriminatory performance, calibration, and incremental value beyond existing clinical and pathological measures. At the therapeutic level, appropriate intervention windows and target patient populations need to be further defined, together with the development of more selective and safer regulatory strategies. Therefore, the true translational value of NLRP3 in SjD may depend not on whether it can be universally inhibited, but on whether precise intervention can be achieved according to cell type, disease stage, and patient phenotype.
